# Effect of Stevioside (*Stevia rebaudiana*) on *Entamoeba histolytica* Trophozoites

**DOI:** 10.3390/pathogens13050373

**Published:** 2024-04-30

**Authors:** Karla Jocelyn Ortega-Carballo, Karla Montserrat Gil-Becerril, Karla Berenice Acosta-Virgen, Sael Casas-Grajales, Pablo Muriel, Víctor Tsutsumi

**Affiliations:** 1 Department of Infectomics and Molecular Pathogenesis, CINVESTAV-IPN, Mexico City 07360, Mexico; karla.ortega@cinvestav.mx (K.J.O.-C.); kgil@cinvestav.mx (K.M.G.-B.); kbacosta@cinvestav.mx (K.B.A.-V.); 2 Laboratory of Experimental Hepatology, Department of Pharmacology, CINVESTAV-IPN, Mexico City 07360, Mexico; sael_89@hotmail.com (S.C.-G.); pmuriel@cinvestav.mx (P.M.)

**Keywords:** amoebiasis, stevioside, adhesion, cytopathic effect, cysteine proteases

## Abstract

Human amoebiasis still represents a major health problem worldwide. Metronidazole has been used as the most common drug to treat the disease; however, it is also known that the drug causes undesirable side effects. This has led to the search for new pharmacological alternatives which include phytochemical compounds with antiamoebic effects. We analyzed the amoebicidal activity of stevioside (STV), a diterpene glycoside present in *Stevia rebaudiana*, on trophozoites of *E. histolytica*. Different concentrations of STV were tested, and an inhibitory concentration of 50% of cell viability (IC_50_) was determined with an exposition of 9.53 mM for 24 h. Trophozoites exposed to STV showed morphological changes evidenced by the decrease in the basic structures related to the movement and adherence to the substrate, as well as ultrastructural features characterized by a loss of regularity on the cell membrane, an increase in cytoplasmic granularity, and an increase in apparent autophagic vacuoles. Also, the decrease in cysteine protease expression and the proteolytic activity of trophozoites to degrade the cell monolayer were analyzed. A histological analysis of hamster livers inoculated with trophozoites and treated with STV showed changes related to the granulomatous reaction of the liver parenchymal tissue. Our results constitute the first report related to the possible use of STV as a therapeutic alternative in amoebiasis.

## 1. Introduction

*Entamoeba histolytica* infection is the third most common cause of death by parasites worldwide after malaria and schistosomiasis infections. Amoebiasis is estimated in over 40 million people around the globe, but only 10% develop a pathologically severe condition, where between 50,000 to 100,000 deaths are reported yearly [1].

The drugs used to treat this infection have been classified based on their site of action. Among these are drugs that act directly on tissues already invaded by trophozoites and those that act in the lumen against non-invasive trophozoites [2]. In symptomatic patients, nitroimidazoles such as metronidazole and tinidazole have been the most used drugs due to their mixed action [3]. However, to this date no drug, including metronidazole, has been shown to affect parasite cysts, which could cause recurrence of the infection [4,5].

Although metronidazole is the most common drug used against amoebiasis [3], adverse side effects such as vomiting, diarrhea, nausea, headache, and a sharp metallic taste in the mouth have been commonly reported [6,7]. Also, metronidazole is known to have teratogenic, mutagenic, and resistance activities on bacterial populations that are present in the human intestine [8]. Currently, research focused on obtaining more efficient and safe therapies that allow us to manage both the intestinal and the extraintestinal amoebic infections is urgently needed. Among the new therapeutic alternatives, compounds present in various plants such as flavonoids, terpenes, tannins, and thiophenes have been proposed in the past, and some of them have shown interesting amoebicidal activity [9].

Up to the present, studies related to the use of stevioside (STV) derivates against parasitic infections have not been reported. Here, we explored the effect of STV on *E. histolytica* trophozoites under experimental conditions. We carried out experiments to show the effect of STV on trophozoite adhesion and virulence, the analysis of the cytopathic effect on infected CaCo2 cells, and the molecular changes in the expression of mRNA levels of cysteine proteases of the parasite. Also, we performed histopathological studies related to the STV effect on amoebic liver abscess (ALA) using an in vivo model.

## 2. Materials and Methods

### 2.1. Amoebic Cultivation

Virulent trophozoites of the *Entamoeba histolytica* HM1-IMSS strain were used in all experiments. Cells were axenically grown at 37 °C in TYI-S-33 medium supplemented with Diamond’s vitamins, 1% penicillin/streptomycin, and 10% adult bovine serum [10]. A culture of parasites was used in the growth logarithmic phase (72 h), harvested by chilling incubated at 4 °C, and centrifuged for 5 min at 350× *g*.

### 2.2. Viability Assays

To determine the effect of the STV Sigma-Aldrich^®^ (St.Louis, MI, USA) on *E. histolytica* trophozoites, viability assays were conducted. Ten thousand trophozoites were seeded in 96-well plates with 300 μL of culture medium for control and experimental conditions. Trophozoites were incubated at 37 °C for 2 h to allow adhesion to the plastic surface. Different concentrations (10, 50, 100, and 150 µM and 5, 7.5, 10, 15, 20, and 25 mM) and exposition times (6, 12, 24, and 48 h) of STV were evaluated, and untreated amoebae were employed as the control. After STV exposure, the culture medium was removed from each well and gently washed with sterile and tempered 1× phosphate-buffered saline (1× PBS). For the determination of cellular viability, the WST-1 reagent Roche^®^ (Basel, Switzerland) was used according to the supplier’s specifications. From the results obtained on cell viability percentage, an inhibitory concentration of 50% of the population (IC_50_) was determined through a dose–response curve with the GraphPad Prism^®^ 6.0 software. Once the IC_50_ was determined, cytotoxicity assays were performed on different cell lines.

### 2.3. Adhesion Assays

Trophozoites were incubated for 24 h at 37 °C with stevioside at a concentration of 9.53 mM leaving trophozoites under normal culture conditions. After the incubation, trophozoites were harvested by cooling at 4 °C for 15 min. Trophozoites (2.5 × 10^5^) were seeded in 24-well plates treated with 100 μg fibronectin. Adhesion assays were carried out at 15, 30, and 60 min. Cell viability was evaluated by trypan blue, and the percentage of adhered cells was determined by a light microscope. 

### 2.4. Immunofluorescence and Confocal Microscopy

Trophozoites (2.5 × 10^5^) were adhered in silanized coverslips for 1 h at 37 °C. STV exposition was performed at 37 °C for 24 h. Samples were fixed with 4% paraformaldehyde for 15 min and permeabilized with 0.2% Triton X-100 for 10 min. After washing, samples were incubated with rhodamine-phalloidin (1:100) for 45 min at room temperature in darkness. Nuclei stains were performed with the Hoechst 33342 reagent (1:1000) at 30 min. Finally, samples were stored at −20 °C until observation by confocal microscopy (Carl Zeiss^®^ LSM 700 microscope, Jena, Germany).

### 2.5. Proteolytic Activity

Crude extracts of trophozoites previously treated with 9.53 mM stevioside for 24 h and untreated trophozoites were obtained for the determination of proteolytic activity by substrate degradation. One million trophozoites for control and STV-treated samples were harvested. For protein extraction, trophozoites were washed in 1× PBS and centrifuged for 5 min at 350× *g* 3 times. The pellet was resuspended in a lysis buffer and incubated on ice for 10 min. The supernatant was recovered after centrifugation for 2 min at 5600× *g* and quantified by the Bradford method. The electrophoretic development of 2 µg of total protein, for both controls and STV-treated samples, was carried out in 15% polyacrylamide gels copolymerized with 0.1% porcine skin gelatin. The gels were washed twice in a 2.5% Triton X-100 solution and incubated overnight into an activation buffer (100 mM Tris-HCl, 10 mM CaCl_2_, pH 7) at 37 °C while stirring. Finally, the gels were washed with tridistilled water and stained with a Coomassie blue solution for 1 h at 37 °C. A densitometric analysis of the bands was performed by the ImageJ^®^ software 1.8.0.

### 2.6. Cytopathic Effect

The measurement of the cytopathic effect was performed according to [11]. Briefly, monolayers of the CaCo2 cell line were cultured, and after the end of the incubation time these cells were interacted with trophozoites previously treated with 9.53 mM stevioside for 24 h and untreated trophozoites for 15, 30, and 60 min. After the interaction times, as many trophozoites were removed as possible, and the cells were washed with cold 1× PBS. Subsequently, the cells were fixed with 2.5% paraformaldehyde. The remains of the monolayers were stained with methylene blue to later be subjected to chemical lysis of the cells via dye extraction and quantified by spectrophotometry at a 660 nm wavelength. Also, CaCo2 cells were attached in silanized coverslips, interacted with trophozoites as mentioned above, fixed with 2.5% paraformaldehyde, and washed with 1× PBS, then stained with Hematoxylin & Eosin (H&E) to be analyzed with a light microscope.

### 2.7. RNA Extraction, cDNA Synthesis, and PCR Studies

Control and treated samples were processed to evaluate the expression of cysteine proteases (cp1, cp2, and cp5). For RNA extraction, the NZYol reagent Nzytech^®^ (Estrada do Paço do Lumiar, Lisboa, Portugal) was used, performed per the manufacturer’s recommendations. Total RNA was resuspended in RNAse-free water and stored at −20 °C until use. Subsequently, cDNA synthesis was performed using the NZY First Strand cDNA Synthesis reverse transcription kit Nzytech^®^ (Estrada do Paço do Lumiar, Lisboa, Portugal), also based on the supplier’s conditions. 

Specific oligonucleotides were used to amplify the sequences corresponding to cysteine protease genes cp1, cp2, and cp5 (NCBI Accession numbers AK418693, AK421545.1, and LC360751.1), and the actin gene (NCBI Accession number M19871.1) (Supplementary Material) was used as an internal control. The PCR assays were carried out using a DNA polymerase kit (NZY^®^Taq II DNA polymerase) Nzytech^®^ (Estrada do Paço do Lumiar, Lisboa, Portugal), and each product was loaded in 2% agarose gels. Densitometric analyses of the obtained bands were conducted by ImageJ^®^ software and plotted using GraphPad Prism^®^ 6.0 software. 

### 2.8. Scanning Electron Microscopy (SEM)

Control and STV-treated trophozoites were adhered to coverslips and fixed in 2.5% glutaraldehyde in a 0.1 M sodium cacodylate buffer and processed as reported in reference [12]. The samples were analyzed with a JEOL-JSM 7100 F scanning electron microscope.

### 2.9. Transmission Electron Microscopy (TEM)

Control and STV-treated trophozoites were fixed with 2.5% glutaraldehyde in a 0.1 M sodium cacodylate buffer and processed as reported in Herrera-Martínez et al. [13]. The samples were analyzed with a JEOL-JEM-1011 transmission electron microscope.

### 2.10. Experimental ALA Induction

Our protocol for animal use was previously approved by the Internal Committee for the Care and Use of Laboratory Animals (CICUAL-CINVESTAV). Eighteen golden hamsters (*Mesocricetus auratus*) were used in two study groups: (1) control and (2) infected. Animals from study group number 2 were infected with 1 × 10^6^ trophozoites previously treated with STV 24 h before intrahepatical inoculation. The lesions were recovered 5 days post-infection. The liver samples obtained were processed for subsequent macroscopic and histopathological analysis by H&E staining. 

## 3. Results

### 3.1. Evaluation of STV Concentrations over Decreased Viability of E. histolytica Trophozoites

**Figure 1:**
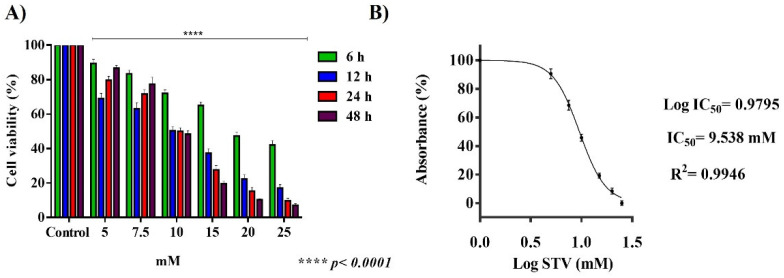
Effect of STV on trophozoites of *E. histolytica* viability. (**A**) mM STV concentrations: 5 and 7.5 mM displayed viability but recovered after 24 h; 10 mM concentration had amoebostatic effect up to 12 at 48 h exposition; higher concentrations than 15 mM showed a toxic effect. (**B**) IC_50_ STV 9.53 mM was concentration used in all experiments to evaluate effect of STV on trophozoites. Values are the means and SD in triplicate of three independent experiments. The date showed a significant difference **** *p* < 0.0001 with respect to control.

The assays carried out with µM concentrations of STV did not show effects on the viability of trophozoites. The exposure of trophozoites to mM concentrations of STV decreased cell viability. Concentrations of 5 and 7.5 mM exhibited a decrease in cellular viability only at 6 and 12 h, while at 24 and 48 h the culture began to recover, proving that neither concentration influences the viability of the amoeba. The STV 10 mM concentration had an amoebostatic effect on trophozoites due to a decreased viability of approximately 20% after 6 h of treatment, while from 12 to 48 h trophozoite viability remained about 50%. Concentrations of 15, 20, and 25 mM displayed a toxic effect against amoebae after 6 h of incubation. The 25 mM concentration had the best effect on the culture at all times evaluated. From that, it is possible to determine that the STV possesses a dose- and time-dependent effect for concentrations from 10 mM to 25 mM (Figure 1A).

The concentration of 10 mM showed an inhibitory effect in approximately 50% of the attached trophozoites at all times evaluated. The IC_50_ of cellular viability after 24 h of incubation with STV was determined to carry out the following experiments. Non-linear regression was carried out, resulting in 9.53 mM of STV as the IC_50_ value that was used in the following experiments (Figure 1B). Once the IC_50_ of stevioside was determined, viability assays were performed in different cell lines. The results obtained showed no differences in the viability of cells treated with stevioside compared to the control.

### 3.2. STV Exposure Decreases the Adhesion Capacity to Fibronectin by E. histolytica Trophozoites

**Figure 2:**
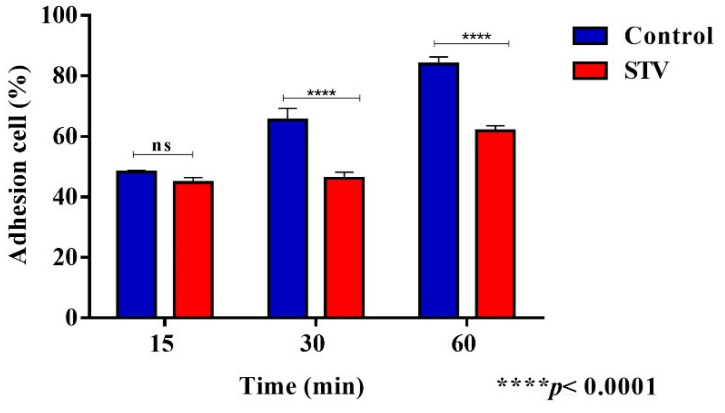
Adhesion capacity after STV exposition. A 15 min exposition with STV did not affect adhesion to fibronectin, while 30 and 60 min of exposition significantly decreased the capacity of trophozoites to adhere to a fibronectin surface. Values are the means and SD in triplicate of three independent experiments. The date showed a significant difference **** *p* < 0.0001 and “ns” indicates that there is no significant difference with respect to the control.

Adhesion assays to fibronectin were carried out by measurements of cell viability of both control and STV-treated trophozoites. The percentage of adhesion at 15 min in both conditions did not show a significant difference, while at 30 and 60 min the percentage of STV-treated trophozoites showed a decrease in the capacity of adhered trophozoites (Figure 2). The adhesion percentage was calculated as the number of initial trophozoites. These results suggest that STV incubation influences the ability of adherence of trophozoites. 

### 3.3. Evaluation of the Effect of STV on the Actin Cytoskeleton

**Figure 3:**
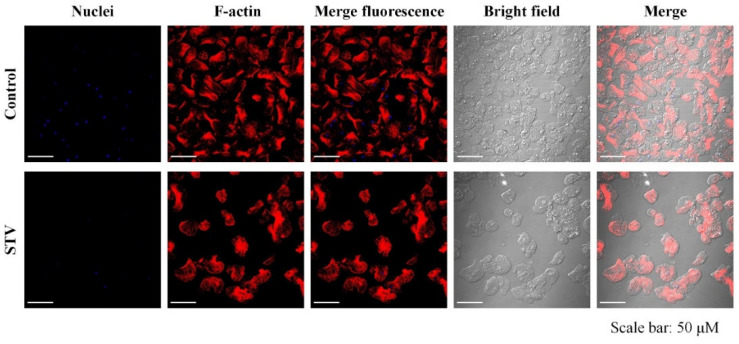
Effect of STV on the actin cytoskeleton. Microscopic analysis of actin fibers displayed in stress fiber number, contractile rings, and nucleation points, suggesting that STV interferes with actin fiber polymerization.

To demonstrate whether STV can alter the structural organization of the actin cytoskeleton, control and STV-treated trophozoites were stained with rhodamine-phalloidin. Non-STV-treated trophozoites displayed nucleation points, stress fibers, and contractile rings. Meanwhile, STV-treated trophozoites showed a lower amount of stress fibers, as seen in Figure 3. These results could suggest a direct effect on the formation of actin-related structures and the rearrangement of actin after incubation with the STV.

### 3.4. Cysteine Protease Activity

**Figure 4:**
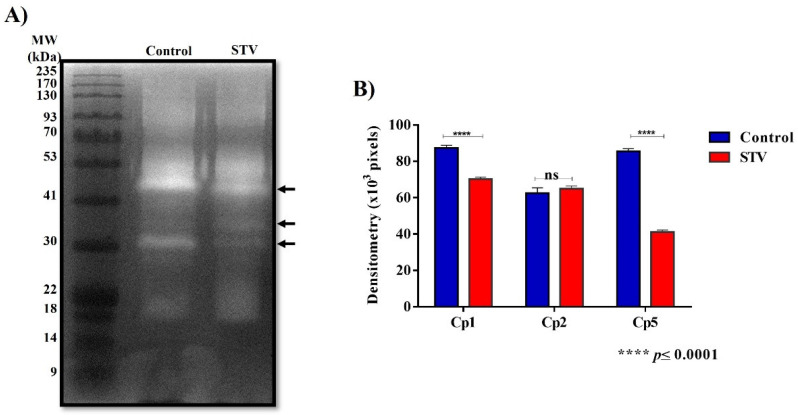
Cysteine protease activity. (**A**) The zymogram obtained with protein extracts from trophozoites treated and not treated with STV shows changes in proteolytic activity at 48, 35, and 29 kDa, corresponding to Cp1, Cp2, and Cp5, respectively (arrows). (**B**) The densitometric analysis of the bands in the zymogram shows that the activity decreases significantly for Cp1 and Cp5 when the trophozoites are treated with STV, and the activity of Cp2 does not show significant changes. Values are the means and SD in triplicate of three independent experiments. The date showed a significant difference **** *p* < 0.0001 and “ns” indicates that there is no significant difference with respect to the control.

After the evidence of physiological changes and actin cytoskeleton structure formation decreased, the proteolytic activity of *E. histolytica* parasites in both control and STV-treated conditions was evaluated in zymograms by the presence of three bands corresponding to Cp1 (48 kDa), Cp2 (35 kDa), and Cp5 (29 kDa), as seen in Figure 4A.

The densitometry measurement of the bands showed a significant difference in the decrease in the proteolytic capacity of Cp1 and Cp5 in STV-treated trophozoites. However, the activity of Cp2 in STV-treated trophozoites did not show significant changes in comparison with the control (Figure 4B).

### 3.5. Cytopathic Effect

**Figure 5:**
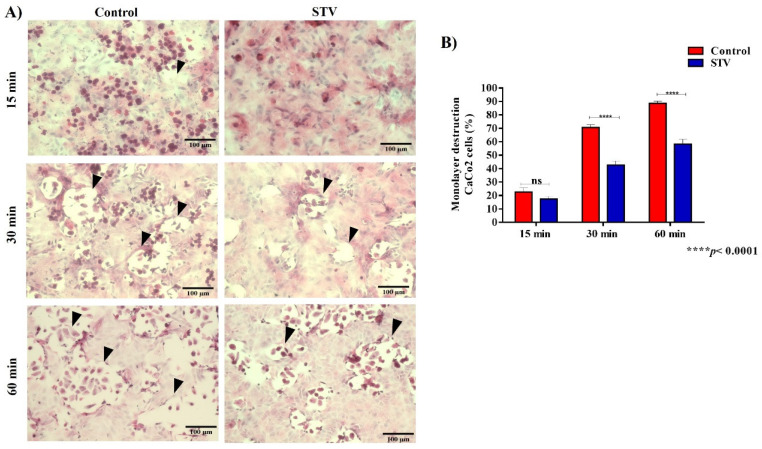
Cytopathic effect of *E. histolytica* trophozoites exposed to STV. (**A**) H&E staining of cell monolayers exposed to trophozoites treated at different times by STV; a greater destructive capacity can be observed in control conditions than with treated amoebae. (**B**) The quantification of methylene blue retained in the remains of monolayers that were exposed to the treated and non-treated trophozoites demonstrates the significant decrease in the cytopathic effect when there was exposure to STV. Arrow heads point to lytic zones. Values are the means and SD in triplicate of three independent experiments. The date showed a significant difference **** *p* < 0.0001 and “ns” indicates that there is no significant difference with respect to the control.

The monolayer destruction capacity was evaluated at different times with the control and STV-treated trophozoites. We observed a smaller amount of trophozoites and decreased lytic areas in the STV-treated condition than the control condition, which suggests that STV affected the capacity of the destruction of monolayers of trophozoites (Figure 5A).

In the analysis of the quantification of the destruction of the cell monolayer, we observed that in the treatment condition, the areas of destruction were smaller at 30 and 60 min compared to the control. Furthermore, no significant changes were observed between either of the conditions at 15 min (Figure 5B).

### 3.6. STV Exposure Decreases the Expression of Cysteine Proteases in E. histolytica

**Figure 6:**
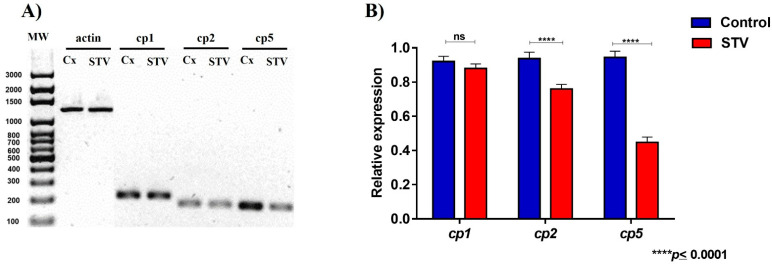
Differential expression of cysteine proteases from *E. histolytica* exposed to STV. (**A**) Amplicons of genes corresponding to actin (endogenous), cp1, cp2, and cp5. (**B**) The histogram of relative expression of cp1, cp2, and cp5 shows that a significant decrease in the expression of cp2 and cp5 was found when the trophozoites were treated with STV; the expression of cp1 was not affected by the treatment. Actin gene expression was used to normalize the data. Values are the means and SD in triplicate of three independent experiments. The date showed a significant difference **** *p* < 0.0001 and “ns” indicates that there is no significant difference with respect to the control.

To determine the effect of STV on the expression of cysteine proteases (cp1, cp2, and cp5), PCR assays were carried out. The expected amplicons for genes of interest in both conditions are actin 1200 bp, cp1 212 bp, cp2 177 bp, and cp5 173 bp (Figure 6A). The results exhibit that STV-treated trophozoites have a lower expression of cp2 and cp5 compared to the control trophozoites. Regarding the expression of cp1, there were no changes between the experimental conditions (Figure 6B).

### 3.7. Morphological Changes in E. histolytica Trophozoites after STV Incubation

**Figure 7:**
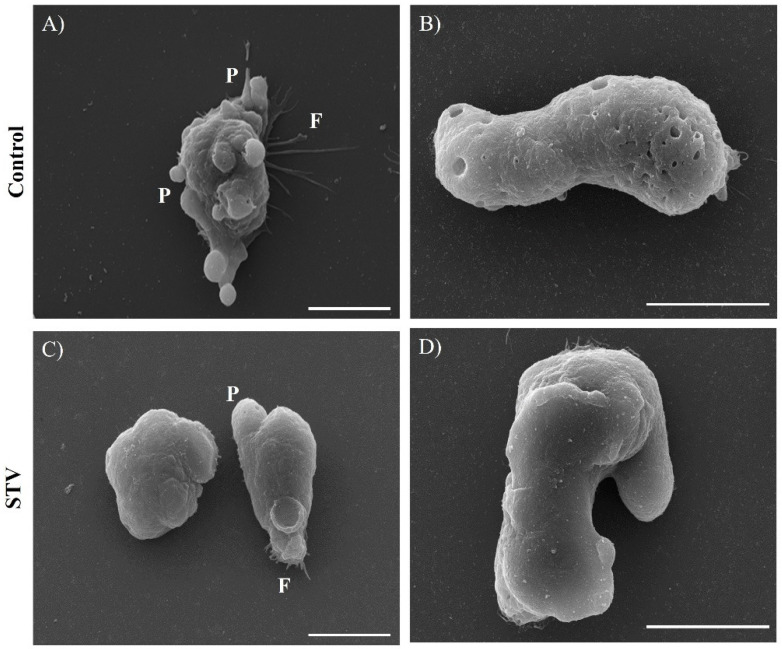
Morphological changes in *E. histolytica* trophozoites upon exposure to STV. (**A**,**B**) Trophozoites without exposure to STV: the emission of pseudopods (P) and filopodia (F) is observed, as well as invaginations of the membrane. (**C**,**D**) Trophozoites treated with STV: the decrease in the emission of filopodia and pseudopods is evident, as well as the decrease in the roughness of the membrane. Bar size: 10 µm.

The normal structure of trophozoites is generally an elongated oval shape, with the emission of filopodia and prominent pseudopodia of various sizes and a rough-looking surface with small circular invaginations corresponding to phagocytic mouths (Figure 7A,B). STV-treated trophozoites showed less formation of pseudopodia and filopodia structures, as well as a less rough-looking surface and a decrease in phagocytic mouth formation (Figure 7C,D). These results can be associated with a reduction in the capacity of the adhesion and consequently possibly in the capacity of invasion of the *E. histolytica* trophozoites after incubation with STV.

### 3.8. STV Incubation Induces Ultrastructural Changes in E. histolytica Trophozoites

**Figure 8:**
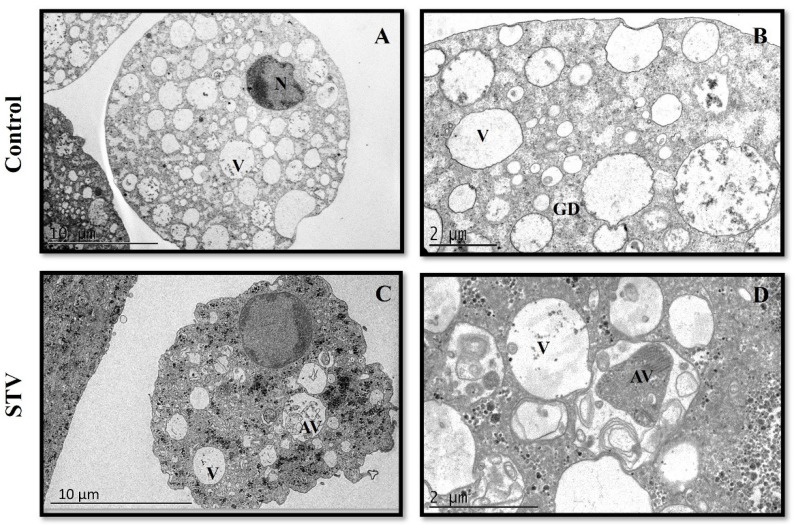
Ultrastructural analysis of trophozoites of *E. histolytica* treated with STV. (**A**,**B**) Control trophozoites displayed a well-defined and uniform plasma membrane; in the cytoplasm, abundant vacuoles of various sizes with spherical appearances were observed, which did not show content, and glycogen deposits scatted in the cytosol and a fully defined nucleus with chromatin of normal appearance located peripherally within the nucleus. (**C**,**D**) Treated STV trophozoites: the cytoplasmic membrane is observed to be interrupted, glycogen stores seem to have increased, vacuoles are abundant, of different sizes with membranous content, and the nucleus and chromatin show no changes.

The analysis of the ultrastructure of normal trophozoites shows a regular cytoplasmic membrane, with glycogen deposits, vacuoles without content, and typic nuclei (Figure 8A,B). The STV-treated parasites showed an irregular-appearing plasma membrane, and a decrease in the number of vacuoles was observed, most of which had cellular debris such as cytoplasmic fragments, granular content, and broken membranes. The cytoplasm was shown to be more electron dense and with an increase in the cellular granularity of unknown origin, while the nucleus showed a similar appearance to the control group (Figure 8C,D). Those results suggest that the presence of membrane and cytoplasmic content inside the vacuoles could be linked to an autophagic process derived from STV exposure.

### 3.9. Effect of STV Treatment on ALA Model

**Figure 9:**
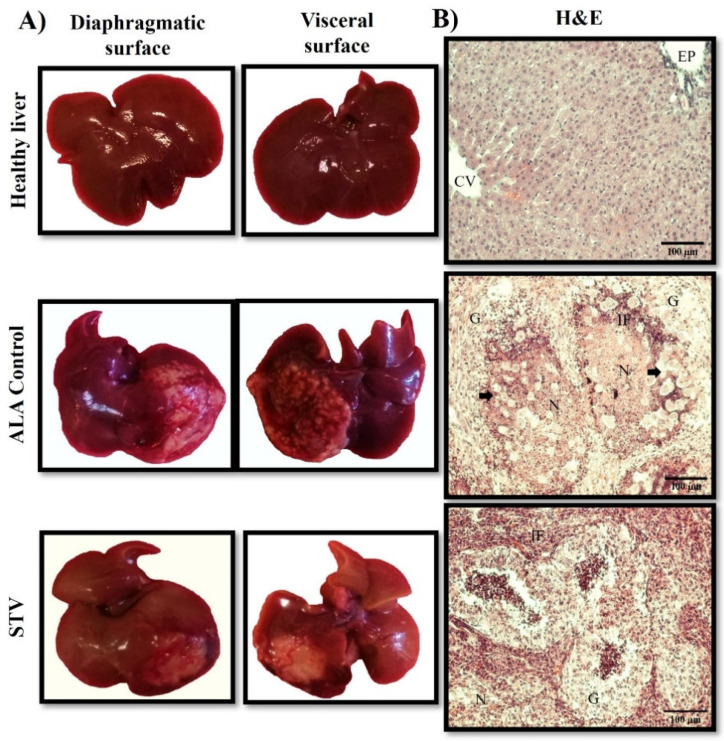
Changes in ALA formation by exposure of *E. histolytica* trophozoites to STV. The controls inoculated with the STV vehicle and with conditioned medium did not show any change (not show) with respect to the healthy control. Macroscopically and microscopically, ALA controls are higher than those generated by STV-treated trophozoites. (**A**) Macroscopically, the ALA does not have a significant difference in size, although the extension of the lesion is less in those treated with STV. (**B**) Microscopically, the parenchyma of the healthy liver is shown when trophozoites are inoculated without treatment with STV; granulomas with very extensive areas of necrosis (N) and inflammatory focus (IF) delimited by epithelioid cells are observed, while granulomas produced by trophozoites exposed to STV generate smaller granulomas (G) delimited by an inflammatory reaction (IR) without a typical granuloma palisade.

To determine the effect of STV on the in vivo virulence of *E. histolytica*, ALAs were allowed to grow for 5 days. The conditions evaluated were the control healthy livers (HLs), conditioned medium of untreated trophozoites (CMUTs), conditioned medium of STV-treated trophozoites (CMSTs), control untreated trophozoites (UTs), and STV-treated trophozoites (STTs). A histological analysis of the different conditions was carried out. In the control animals, macroscopic normal characteristics of the liver were observed; the samples treated with a conditioned medium for both conditions (CMUT and CMST) also displayed macroscopic conditions characteristic of a normal liver. On the other hand, a macroscopic analysis of the UT livers showed that they developed ALAs with an average of liver damage of 42%, which was mainly observed in the diaphragmatic or visceral face of lobules. STTs developed ALAs with 35% liver damage; these lesions, as in the untreated trophozoites, could be observed both on the diaphragmatic and visceral face (Figure 9A). Although the percentages of lesions were determined by registering the weight of dissected lesions to the total liver weight, no statistically significant differences were found between the STV-treated and control groups. 

Representative fragments of the lesions were analyzed by histopathology. For the HL condition, a characteristic parenchyma was observed, with the presence of a centrilobular vein (CV) and a portal space (SP) surrounded by cords of hepatocytes and sinusoids, without any histological alteration. Regarding the samples of the two conditioned media, the cellular architecture was normal for healthy liver tissue. On the other hand, in the lesions produced by UTs, a well-defined granuloma (G) delimited by palisade-shaped epithelioid cells was observed, surrounded by necrotic areas (N) extending throughout much of the liver parenchyma, as well as the presence of inflammatory focus (IF) and trophozoites in the periphery of the granulomatous lesions. In the lesions produced by STTs, a strong IF was observed, followed by the formation of multiple smaller Gs delimited mostly by inflammatory cells; however, a typical granuloma palisade was not observed, and the trophozoites present in the lesion areas appeared less commonly when compared to those observed in the control conditions (Figure 9B).

## 4. Discussion

Plant-derived compounds have represented an important source for the search for new therapeutic alternatives in infectious diseases. Some studies have reported the antiamoebic effectiveness of plant extracts and specific molecules such as terpenes and thiophenes [14,15]. 

Also, molecules with antiamoebic activity have been isolated from different members of the Asteraceae family, used mainly in the treatment of diarrhea, laryngitis, and body pain, where the active metabolites are flavonoids and terpenes [16]. *Stevia rebaudiana* is also a plant of the Asteraceae family, with diterpene glycosides such as STV and rebaudioside A, which are the ones with the highest contents in the leaves of the plant [17,18]. STV can induce apoptosis in various cell lines through the production of reactive oxygen species, which has been evidenced by morphological changes in the size and shape of cells, fragmentation of nuclei, and biochemical changes such as the externalization of phosphatidylserine [19,20,21].

The beneficial effects of STV, such as its anti-inflammatory, anti-hypertensive, anti-tumoral, and antiviral properties, have been reported [17,19,20,21,22,23]. However, up to the present, an anti-parasitic effect has not been described.

In this work, we exposed *E. histolytica* trophozoites to different µM and mM concentrations of STV, and trophozoites exposed to μM concentrations of STV did not present an affectation in their viability. Our results revealed that the amoebas exposed to mM concentrations showed a lethal effect depending on the dose and time exposition (Figure 1A); also, IC_50_ was determinate, resulting in 9.53 mM at 24 h of exposition being the condition that was used in the following experimental procedures (Figure 1B).

Adhesion to the extracellular matrix regulates the organization of the actin cytoskeleton, such as the formation of lamellipodia and filopodia during the early stages up to the formation of focal adhesions and stress fibers in fully adherent cells [24]. It has been shown that natural compounds derived from medicinal plants such as kaempferol can deregulate proteins related to the actin cytoskeleton, such as myosin II and cortexilin II, resulting in the evolution of invasion mechanisms [25]. In addition, it has been reported that reactive oxygen species can affect cytoskeletal functions [14], and that the deregulation of Rho family GTPases such as Rac and Cdc42 could alter the formation of stress fibers [26,27]. With the assays of adherence to a fibronectin surface that was carried out with STV-treated trophozoites, a decrease in adherence could be observed (Figure 2). Also, changes in the actin cytoskeleton generated by the STV were evaluated, where we could observe structures such as stress fibers, contractile rings, and nucleation points in the control trophozoites, while the STV-treated trophozoites showed an increase in actin polymerization without the formation of stress fibers and focal adhesions, which could be associated with a decrease in the adhesion capacity of the amoeba (Figure 3). A comparative analysis of the morphology and molecular structure of the actin cytoskeleton between *E. histolytica* and *E. dispar* has been previously reported, and the authors evaluated the adhesion capacity of *E. histolytica* that firmly adheres to fibronectin, forming a very narrow space between the junction of the plasma membrane with the substrate [12].

In amoebae, the differences in the structuring of the actin cytoskeleton are related to the invasion capacity of *E. histolytica* compared to the non-pathogenic species, *E. dispar*. It has been reported that the actin of *E. histolytica* adhered to the substrate in an organized manner with well-defined structures as focal adhesions, while in *E. dispar*, trophozoites present an irregular and distributed actin polymerization throughout the cytoplasmic compartment [12]. On the whole, our results show a direct effect on the ability of the parasite to bind to a substrate and cause morphological changes; therefore, exposing trophozoites to STV could eventually prevent their establishment in the host’s intestine and even reduce the possibility of extraintestinal complications by not having the necessary faculties to invade tissues. This is based on the rearrangement of the actin cytoskeleton evidenced by the increase in polymerized actin, which decreases the amoeba’s adhesion capacity and affects the pathogenic mechanisms of this parasite (Figure 3). 

The proteolytic activity was analyzed by obtaining zymograms where three bands were identified for both conditions, corresponding to Cp1, Cp2, and Cp5. A significant decrease in the proteolytic activity of Cp1 and Cp5 was demonstrated in the treated extracts, while the activity of Cp2 was not affected by the treatment (Figure 4A). Cysteine proteases are proteolytic enzymes secreted by the amoeba within its microenvironment, constituting important virulence factors, and are considered essential in the ability of this parasite to destroy human tissues and to degrade different components of the extracellular matrix, such as fibronectin, laminin, and collagen [28,29,30]. Previously, it has been reported that the trophozoites that overexpress this Cp2 do not show differences in their erythrophagocytosis capacity, the development of ALA, or the decrease in the cytopathic effect, while the expression of Cp1 and Cp5 plays an important role in its pathogenicity since when they have inhibited the cytopathic effect, the development of ALA is affected [28]. Our results show that STV can affect trophozoite virulence via a significant decrease in Cp1 and Cp5 activity (Figure 4B).

In vitro assays have shown that trophozoites of *E. histolytica* destroy monolayers of mammalian cells, such as Chinese hamster ovary cells. Cytopathic effects can be blocked by specific inhibitors of cysteine proteases, such as E-64 [28]. In our results of proteolytic activity, we observed a decrease in Cp1 and Cp5 (Figure 4); to evaluate the destructive capacity of the amoeba, we performed an interaction of trophozoites treated and untreated with STV, where we verified that the decrease in the proteolytic activity of these cysteine proteases decreases the amoeba’s ability to destroy cell monolayers (Figure 5).

Quantitative studies of the expression of the genes of amoebae cysteine proteases have shown that cp1 is one of the most expressed and released cysteine proteinases in cultured trophozoites. Furthermore, if cp1 is increased, it can cleave physiological substrates as components host immune responses. Also, in vivo assays showed that specific inhibitors of cp1 block invasion in the human colon [31]. In another study, in a mouse model of amoebic colitis, cp1 expression increased almost two-fold after the invasion, whereas cp5 expression did not [32]. 

In this study, was analyzed the expression of cp1, cp2, and cp5 in STV-treated and untreated trophozoites. We observed that the relative expression showed a decrease in cp2 and cp5; however, cp1 did not show differences compared to the control, and this allows us to observe that STV affects the expression mechanisms of cysteine proteases in trophozoites of *E. histolytica* (Figure 6).

Trophozoites exposed to the IC_50_ STV also presented morphological changes; these were manifested by SEM, where we observed fewer pseudopods and filopodia formations compared with untreated trophozoites (Figure 7). These structures are associated with the amoeba’s ability to invade, and these results suggest that STV exposition decreases the invasion capacity of the exposed trophozoites.

Otherwise, using TEM, interesting ultrastructural changes were observed in trophozoites treated with STV, such as a loss of membrane regularity, increased granularity, a decrease in the number of vacuoles, and the production of vacuoles with granular, membrane, and cytoplasmic content, as compared with the non-treated parasites (Figure 8). Similar effects at the ultrastructural level have been previously reported with various molecules tested as amebicides, such as the sesquiterpene linearolactone, which causes changes at the ultrastructural level evidenced by the increase in glycogen deposits and vacuoles with greater granular content, which could be associated with the inhibition of the glycogen metabolism [15].

The pathogenicity of this parasite is based on motility, adhesion, and cell lysis, processes for which the actin-rich cytoskeleton is responsible. Mechanisms of invasion are driven by adhesion to the target cell, where actin reorganization is indispensable [33]. Our results suggest that morphological and ultrastructural alterations produced by STV treatment decrease mobility and adherence ability due to alterations in the actin cytoskeleton (Figure 7 and Figure 8).

In the in vivo virulence assays, macroscopically speaking, no significant changes were observed in the size of the lesion produced after STV treatment at 5 days post-infection. However, histological sections showed that STV-treated trophozoites provoked a bigger inflammatory response, areas of necrosis like the control, and the formation of multiple granulomas of smaller size than the control, delimited mainly by inflammatory and non-epithelioid palisade cells. In the non-treated trophozoites, large areas of necrosis and inflammatory infiltrate and the formation of extended granulomas in the liver parenchyma, delimited by palisade epithelioid cells, were observed. Although no macroscopic difference was observed in the ALAs generated by trophozoites without treatment and those treated with STV, the histopathological analysis suggests a decrease in the progression of damage caused by trophozoites treated with STV (Figure 9).

Our results represent the first study using STV derived from the plant *Stevia rebaudiana* on *E. histolytica* trophozoites. However, further in vivo studies are important to evaluate the role of STV in lesion development; therefore, a complete and integrative analysis is needed to determine if STV works as an effective treatment for amoebiasis. Future analyses will include that of STV in strains of *E. histolytica* with different capacities for virulence and the study of the administration of STV to hamsters before the inoculation of amoebas in the live model and during the development of the lesion to determine the effect of STV in the development of ALAs.

## Data Availability

The data presented in this study are available upon request from the corresponding author.

## Supplementary Materials

The following supporting information can be downloaded at: https://www.mdpi.com/article/10.3390/pathogens13050373/s1, Table S1: Gene amplification condition.
