# Correction: Ortega-Carballo et al. Effect of Stevioside (*Stevia rebaudiana*) on *Entamoeba histolytica* Trophozoites. *Pathogens* 2024, *13*, 373

**DOI:** 10.3390/pathogens14070653

**Published:** 2025-07-01

**Authors:** Karla Jocelyn Ortega-Carballo, Karla Montserrat Gil-Becerril, Karla Berenice Acosta-Virgen, Sael Casas-Grajales, Pablo Muriel, Víctor Tsutsumi

**Affiliations:** 1 Department of Infectomics and Molecular Pathogenesis, CINVESTAV-IPN, Mexico City 07360, Mexico; karla.ortega@cinvestav.mx (K.J.O.-C.);; 2 Laboratory of Experimental Hepatology, Department of Pharmacology, CINVESTAV-IPN, Mexico City 07360, Mexico

In the original publication [1], there was a mistake in Figure 6 as published. The figure shown in Section 3.6 of the results does not correspond to the data reported in this section. The corrected version of Figure 6 appears below.

The authors state that the scientific conclusions are unaffected. This correction was approved by the Academic Editor. The original publication has also been updated.

## Figures and Tables

**Figure 6:**
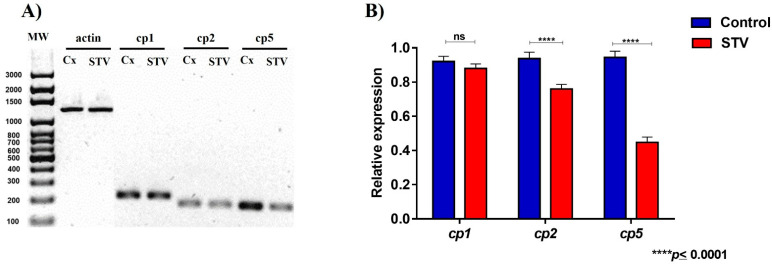
Differential expression of cysteine proteases from *E. histolytica* exposed to STV. (**A**) Amplicons of genes corresponding to actin (endogenous), cp1, cp2, and cp5. (**B**) The histogram of relative expression of cp1, cp2, and cp5 shows that a significant decrease in the expression of cp2 and cp5 was found when the trophozoites were treated with STV; the expression of cp1 was not affected by the treatment. Actin gene expression was used to normalize the data. Values are the means and SD in triplicate of three independent experiments. The date showed a significant difference **** *p* < 0.0001 and “ns” indicates that there is no significant difference with respect to the control.
