# Supplementary material for: Effect of Stevioside (Stevia rebaudiana) on Entamoeba histolytica Trophozoites
Source: Pathogens. 2024 Apr 30;13(5):373. doi: 10.3390/pathogens13050373 (PMC11123825; doi:10.3390/pathogens13050373)
Supplement: Supplementary file 1 [file pathogens-13-00373-s001.zip › pathogens-2950466-supplementary.pdf]

Supplementary material. Gene amplification condition.

Gen	Sequence	Tm (ºC)	Product (pb)	$MgCl_2$ (mM)
**cp1**	F: 5´ACAGCAGTTGAGTCACTCCG 3´<br>R: 5´ACTGCTTTGGGTGCTTGGAT 3´	55	212	1
**cp2**	F: 5´TGGACCATTTGCTGCTATGA 3´<br>R: 5´TAACATGATCCGCATTGTGC 3´	52	177	1
**cp5**	F: 5´CGCTGCTATTGATGCTTCTG 3´<br>R: 5´CTCCCCAAATAGTTCCCCAT 3´	52	173	1.5
**actin**	F: 5´GCACTTGTTGTGGATCCTGGA3´<br>R: 5´CTCGAGTTAGAAGCATTTTCT3´	55	1200	1.5
